# Binding of Y-P30 to Syndecan 2/3 Regulates the Nuclear Localization of CASK

**DOI:** 10.1371/journal.pone.0085924

**Published:** 2014-02-03

**Authors:** Peter Landgraf, Marina Mikhaylova, Tamar Macharadze, Corinna Borutzki, Ana-Claudia Zenclussen, Petra Wahle, Michael R. Kreutz

**Affiliations:** 1 Research Group Neuroplasticity, Leibniz Institute for Neurobiology, Magdeburg, Germany; 2 Department of Experimental Obstetrics and Gynaecology, Medical Faculty, Otto-von-Guericke University, Magdeburg, Germany; 3 Developmental Neurobiology, Faculty of Biology and Biotechnology, Ruhr University, Bochum, Germany; University of Louisville, United States of America

## Abstract

The survival promoting peptide Y-P30 has documented neuroprotective effects as well as cell survival and neurite outgrowth promoting activity *in vitro* and *in vivo*. Previous work has shown that multimerization of the peptide with pleiotrophin (PTN) and subsequent binding to syndecan (SDC) -2 and -3 is involved in its neuritogenic effects. In this study we show that Y-P30 application regulates the nuclear localization of the SDC binding partner Calcium/calmodulin-dependent serine kinase (CASK) in neuronal primary cultures during development. In early development at day in vitro (DIV) 8 when mainly SDC-3 is expressed supplementation of the culture medium with Y-P30 reduces nuclear CASK levels whereas it has the opposite effect at DIV 18 when SDC-2 is the dominant isoform. In the nucleus CASK regulates gene expression via its association with the T-box transcription factor T-brain-1 (Tbr-1) and we indeed found that gene expression of downstream targets of this complex, like the GluN2B NMDA-receptor, exhibits a corresponding down- or up-regulation at the mRNA level. The differential effect of Y-P30 on the nuclear localization of CASK correlates with its ability to induce shedding of the ectodomain of SDC-2 but not -3. shRNA knockdown of SDC-2 at DIV 18 and SDC-3 at DIV 8 completely abolished the effect of Y-P30 supplementation on nuclear CASK levels. During early development a protein knockdown of SDC-3 also attenuated the effect of Y-P30 on axon outgrowth. Taken together these data suggest that Y-P30 can control the nuclear localization of CASK in a SDC-dependent manner.

## Introduction

In recent years a number of bioactive peptides have been identified that affect neurite outgrowth and provide neuroprotection. The survival promoting peptide Y-P30 is one of these factors [1] and was identified later on also as a peptide that promotes the survival of organotypic thalamic cultures [2]. Y-P30 is a 30 amino-acid peptide that derives from a larger precursor that also includes the anti-bacterial peptide dermcidin [3]. The dermcidin gene was not identified in the genome of rodents [4], albeit peptides of the precursor have been identified in various proteomic screens in rodent tissues [2], [5]–[10]. Despite the unclear status of the expression of the peptide in non-primate species it is well documented that Y-P30 has profound neuroprotective, cell migration and neurite outgrowth promoting effects [1], [2], [11]–[14]. The neurite outgrowth promoting activity of Y-P30 appears to depend on binding to the trophic factor pleiotrophin (PTN) and the cell adhesion molecule syndecan (SDC), which results in a trimeric signaling complex that following neuronal polarization selectively stimulates the growth of axons [11]. Multimerization of Y-P30 and PTN may result in larger SDC clusters, which in turn might be involved in neuronal signaling [11].

**Figure 1:**
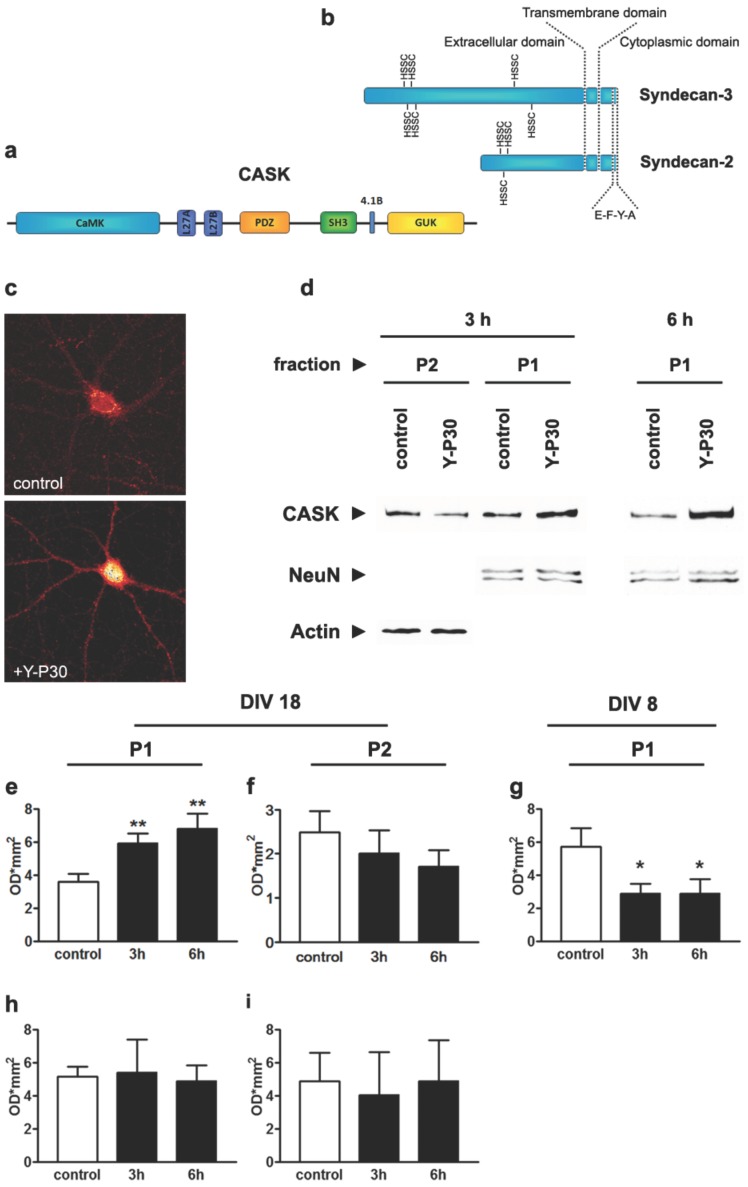
Y-P30 regulates the nuclear distribution of CASK in primary cortical neurons. (a) CASK is a multidomain scaffolding protein. While its GUK-domain interacts with the transcription factor Tbr1, the PDZ domain is responsible for binding to the C-terminus of SDC. (b) Essential for this binding are the final four amino acids that are conserved within all SDC. The binding of Y-P30 is mediated via heparan sulfate side chains (HSSC). (c) Supplementation of primary cortical neurons with Y-P30 at DIV 18 leads to the accumulation of CASK in the nucleus as visualized in confocal images following CASK antibody staining. Scale bar is 20 µm. (d) For quantitative analysis fractions of nuclei (P1) and remaining cellular components (P2) were prepared and analysed with quantitative immunoblotting. In order to normalize the relative amounts of CASK, the P1 fraction was analysed in relation to the NeuN signal, the P2 fraction to the Actin signal. (e) In mature primary cortical cultures (DIV 18) supplementation with Y-P30 leads to a significant increase of the CASK concentration in P1 fractions after 3 and 6 h whereas the CASK concentration in P2 declines albeit not significantly at the same time points. (g) Interestingly, a decrease of the CASK concentration in P1 fractions of young neurons (DIV 8) 3 and 6 h after supplementation with Y-P30 was observed. (h, i) The effect of Y-P30 on the nuclear localization of CASK was abolished in mature neurons (DIV18) treated with Heparitinase I and Chondroitinase A, B, C, cleaving the HSSCs. Note that all fractionation assays were done in the presence of 7.5 µM anisomycin. For Western-blots 20 µg of protein were loaded per lane. Relative concentrations of CASK were analysed by measuring the optical density of the respective signal and normalized as described above. Black boxes indicate treatment with Y-P30. N = 4–11 in each group. ** p<0.001, * p<0.05.

SDC-2 and -3 can associate via their C-terminal binding motif with the MAGUK family member Calcium/calmodulin-dependent serine kinase (CASK) [15], [16]. The interaction occurs with the post-synaptic density 95/discs large/zonula occludens-1 (PDZ) domain of CASK and requires the C-terminal PDZ-binding motif of both SDC (Fig. 1a+b). It has been previously reported that CASK might be imported in the nucleus in early development [17]. The underlying mechanisms are unknown. But it was shown that SDC-2 and -3 play a role during neuronal development for the localization of CASK either in the nucleus or at the cell membrane [15]–[17]. Nuclear CASK regulates gene expression via association with the transcription factor T-box transcription factor T-brain-1 (TBR-1), which binds to a T-box motif in many developmentally regulated genes like reelin, a secreted glycoprotein important for cell migration, cortical wiring and stabilizing cortical cytoarchitecture [18], [19], or the GluN2B NMDA-receptor subunit that determines synaptic maturation [20]. We therefore asked in this study whether the neurite outgrowth promoting activity during development might be related to the regulation of nucleocytoplasmic shuttling of CASK.

## Materials and Methods

### Ethics Statement

In the present experiments, animal care and procedures were approved and conducted under established standards of the German federal state of Sachsen-Anhalt, Germany in accordance with the European Communities Council Directive (86/609/EEC).

### Preparation of dissociated primary cell cultures from hippocampus and cortex

Neuronal primary cultures were prepared as described previously [21]. In brief, rat cortices and hippocampi were prepared from embryos (Long Evans rats) at stage E19 and subsequently transferred into ice cold Hanks Balanced Salt Solution without Mg^2+^/Ca^2+^ (HBSS, Gibco, Karlsruhe, Germany). After triple washing with 5 ml HBSS, 2.0 ml HBSS containing 0.5% trypsin (Sigma) was added, followed by incubation for 20 minutes at 37°C. The tissue was then washed 5 times with 5 ml HBSS and finally transferred into 2 ml tubes with HBSS, containing 0.01% DNAse-I (Invitrogen). For dissociation the respective tissue was pressed three times slowly through a 0.9 mm-gauged needle followed by 3 passages through a 0.45 mm-gauged needle. The remaining cell suspension was poured through a 70 µm cell strainer (BD Biosciences, San Jose, USA) into a 50 ml tube and filled up with 18 ml Dulbecco's modified Eagle Medium (DMEM; Gibco), containing 10% FCS, 2 mM glutamine and 1% Penicillin/Streptavidin (DMEM+). After estimating cell quantity, the suspension was diluted with DMEM+ according to the experimental required density. For axonal growth assays and morphological analyses the cells were plated in a density of 10.000/cm^2^, for biochemical assays in a density of 80.000/cm^2^ in culture flasks. Y-P30 was applied in a final concentration of 6 µg/ml to the bath medium for the indicated periods of time.

### Immunocytochemistry

Cell cultures were fixed with 4% PFA (Merck, Darmstadt, Germany), washed 3 times with 10 mM PBS and subsequently pre-incubated for 90 min at 4°C in blocking solution (10 mM PBS, 10% normal horse serum (NHS; PAA Laboratories, Pasching, Austria), 2% bovine serum albumin (BSA, Sigma, Taufkirchen, Germany), 5% sucrose (Merck), 0.3% Triton X100 (Sigma). Afterwards cells were incubated at 4°C over night in blocking solution containing the primary antibodies in the respective dilutions. The cultures were then washed 3 times with 10 mM PBS containing 0.3% Triton X100 and incubated with secondary antibody for 2 hours. Finally cells were washed 2 times with 10 mM PBS containing 0.3% Triton X100, once with 10 mM PBS and subsequently covered with Moviol (Merck, Darmstadt, Germany). The dilutions for primary antibodies were: mouse anti-CASK (Acris, Germany) 1∶1000, mouse anti-Tau (Chemicon/Millipore, Germany) 1∶1000, mouse anti-MAP2 (Sigma, Taufkirchen, Germany), 1∶1000. As secondary antibody Cy3-conjugated goat-anti-mouse IgG (Dianova, Germany) 1∶1500 were used.

### Microscopic evaluation and morphological analysis

Images were taken using a Zeiss-Axioplan II imaging fluorescent microscope (Zeiss, Jena, Gemany), Spot RT camera and Meta View software (Visitron Systems, Puchheim, Germany). Morphological analysis of neurons was carried out with ImageJ, a Java-based image-processing program developed at the National Institutes of Health (USA; http://rsb.info.nih.gov/ij/index.html), as reported previously [22].

### Expression of GFP-tagged Syndecan-2 and 3 in COS-7 cells

COS-7 cells (Cell Lines Service GmbH, Eppelheim, Germany) were transfected with Lipofect 2000 (Invitrogen, Karlsruhe, Germany) according to the manufacturers instructions. Transfection efficiencies and expression rates were monitored by fluorescent microscopy. At the appropriate time points the cells were scraped with 10 ml ice-cold TBS and subsequently centrifuged for 5 minutes at 1000×g and 4°C. Afterwards the cell pellets were washed in TBS and centrifuged again. The remaining cell pellets were processed directly or shock frozen in liquid nitrogen.

### SDS-PAGE and Western-Blot experiments

For SDS-PAGE or immunoblot experiments protein fractions were solubilized with 4×SDS sample buffer (250 mM Tris-HCl, pH 6.8, 1% SDS, 40% glycerol, 20% ß-mercaptoethanol, 0.004% brome phenol blue) and cooked for five minutes. Afterwards they were separated on 5–20% SDS-Polyacrylamide gradient gels (except for SDC-3 where 4–12% gradient gels were used) and subsequently transferred to nitrocellulose membranes (90 min, 200 mA). The transfer buffer contained 25 mM Tris, 192 mM glycine, 0.02% SDS and 20% methanol. After blotting the membranes were blocked with 5% dry milk and 0.1% Tween 20 in 1×TBS for 2 hours. Subsequently the membranes were incubated at 4°C over night with primary antibodies in the respective concentration in 1×TBS containing 0.1% Tween 20. Thereafter the blots were incubated for 90 minutes at room temperature with HRP-conjugated secondary antibodies (1∶4000) and finally developed using ECL-Films. Phosphoprotein purification was done according to manufacturers instructions (Qiagen, Hilden, Germany). Antibodies against SDC-3 (Abnova (PAB9044); dilution 1∶1500) and GluN2B (NeuroMab, Cat.No. 75–101; dilution 1∶1000), mouse anti-CASK (Acris, Germany; dilution 1∶1000) were used for immunoblotting.

### Fractionation of nuclei from primary cortical cultures

Nuclear fractions were prepared essentially according to a modified and shortened protocol of Reyes et al. [23]. In brief, after washing with 5 ml 1×TBS, pH 7.8, primary cortical cultures of one flask (1.8 Mio cells/flask) were scraped with 1 ml ice cold HNB (0.5 M sucrose, 15 mM Tris/HCl pH 7.5, 60 mM KCL, 0.25 mM EDTA (pH 8), 0.125 mM EGTA (pH 8), 0.5 mM Spermidine, 0.15 mM Spermin, EDTA-free Complete Protease-Inhibitor-Cocktail (Roche)) and centrifuged for 5 min at 500×g at 4°C. The remaining pellet was re-suspended in 750 µl HBN and homogenized with 12 strokes at 900 rpm in a Potter S homogenizer. The homogenate was transferred into 1.5 ml Eppendorf tubes and supplemented with 375 µl HBN containing 1% Nonidet P40 (Sigma). After incubation for 5 min on ice the homogenate was spun for 4 min at 1000×g. The resulting pellet (P1) contained the cell nuclei and was directly solubilized and boiled for 5 min at 95°C with 100 µl SDS-sample buffer. Remaining cell components, in particular the membrane fractions were precipitated via a second centrifugation step at 20800×g for 20 min (P2) and solubilized in 60 µl SDS-sample buffer as described above.

### Precipitation of extracellular proteins from COS-7 cells and subcellular membrane fractionation

The culture medium (DMEM+) of COS-7 cells, expressing Syndecan-2int.myc-GFP, was completely removed and after washing with pre-warmed HBSS replaced with 5 ml HBSS+ containing 20 µg/ml Brefeldin A (Sigma, Taufkirchen, Germany). After 30 min, the cells were supplemented with 20 µM Y-P30 (final concentration) or with the appropriate volume of 5 mM Tris-HCl, pH 7.4 as a control. In order to inhibit matrix metalloproteinase activity either 50 nM GM6001 (Calbiochem, Darmstadt, Germany) or 20 nM of the specific MMP9/13-Inhibitor I (Calbiochem) were added. Three hours later the HBSS-medium was harvested and centrifuged at 3000×g. 5 ml of the remaining supernatant were mixed with 20 ml freezing ethanol and incubated at −20°C over night. On the next day, the precipitated proteins were centrifuged at 4°C and 10.000 rpm for 10 min and the resulting pellet was washed twice with 15 ml 80% ethanol (−20°C). Residual ethanol was removed by lyophilizing the pellets for 5 min. Afterwards the protein pellet was dissolved for 3 h at 4°C in 100 µl ultrapure water containing 2×EDTA-free complete proteinase inhibitor (PI, Roche) and finally solubilized by adding 100 µl 2×SDS sample buffer and boiling for 5 min at 95°C.

For subcellular membrane fractionation corresponding pellets from COS-7 cells were re-suspended in 800 µl HOM-buffer (5 mM Hepes, pH 7.4, 0.32 M sucrose, PI) and subsequently homogenized with 15 strokes at 900 rpm in a Potter S homogenizer. The resulting homogenates were centrifuged in 1.5 ml Eppendorf tubes for 10 min at 1000×g at 4°C. Afterwards, the supernatants (S1) were transferred in fresh tubes and kept on ice. The pellets were re-suspended again and the procedure repeated. In the next both supernatants of the respective sample (S1 and S1′) were combined and spun for 30 min at 20.000×g. The resulting pellets were again re-suspended in 800 µl HOM-buffer, homogenized with 12 strokes at 900 rpm and centrifuged for 30 min at 20.000×g. The obtained pellets represent a crude membrane fraction and were re-suspended in 800 µl 5 mM Tris, pH 8.1, containing 0.32 M sucrose and PI and were subsequently loaded on top of a 1.1 M/1.4 M sucrose step gradient and finally centrifuged for 2 h at 85.000×g and 4°C in a ultra centrifuge. Subsequently, the membrane-containing fraction was transferred into a fresh tube and the sucrose concentration adjusted to 0.32 M with 5 mM Tris buffer+PI, pH 8.0. In a final centrifugation step the membrane fractions were spun for 1 h at 150.000×g at 4°C. The remaining pellets, containing highly enriched cellular membranes, were directly solubilized in 1×SDS sample buffer and boiled for 5 min at 95°C.

### mRNA purification and reverse transcription

For Real-Time PCR experiments two million cells/culture-flasks of primary cortical cultures were harvested at the appropriate time-points and treatments. After washing twice with 5 ml ice cold 1×PBS, pH 7.4, cells were scraped in 1 ml of the same buffer and afterwards centrifuged for 5 min at 1000×g and 4°C. The resulting pellets were re-suspended in 0.6 ml OL1-buffer, containing 0.43 M ß-ME, from the Oligotex mRNA purification kit (Qiagen, Hilden, Germany) that was used for mRNA purification. For efficient cell disruption, the suspension was centrifuged for two minutes at maximum speed through QIAshredder homogenizers (Qiagen). The obtained mRNAs were immediately quantified and reverse transcribed into cDNA, using the Sensiscript RT kit (Qiagen) according to the manufacturers protocol. Reverse primer OdT18 (Promega) and random nanomers (Sigma, Taufkirchen, Germany) were used in separate reaction mixes. The success of the reverse transcription was verified via PCR reaction using specific primers for GAP-DH (not shown). Finally the corresponding cDNAs of one sample were combined and used as one template.

### Quantitative Real-Time PCR (qRT-PCR)

Quantitative Real-Time PCR was performed using the LightCycler 1.5 Instrument from Roche (Roche Applied Science, Mannheim, Germany). For the reaction mix the LightCycler TaqMan Master kit (Roche) was used according to the manufacturers instructions. Primer and probe reagents were ready-made reagents using FAM-dye (Pre-designed TaqMan Assay Reagents; Applied Biosystems) with the following assay- and corresponding GenBank accession numbers: Hprt1 Rn01527840_m1/NM_012583.2, Reelin Rn00589609_m1/NM_080394.2. For NR2B analysis the reagent was designed from the Custom TaqMan Gene Expression Assay Service (Applied Biosystems) with following primer- and probe sequences: GluN2B-237s fw 5′-CAAGCCTGGCATGGTCTTCT-3′, rev 5′-GGATTGGCGCTCCTCTATGG-3′, probe 5′-FAM-CCATCAGCAGAGGTATCT-NFQ-3′ (M91562.1). qRT-PCR reactions were started with an initial denaturation step for 10 min at 95°C, followed by 45 cycles of 95°C for 10 sec, 60°C for 60 sec and 72°C for 1 sec. Relative amounts of Reelin and GluN2B had been normalized with Hprt for mRNA amount variations. The mRNA expression is presented as the change of relative quantities and was analyzed using the 2-ΔΔCt method [24].

### Expression- and knock down constructs

Full-length cDNAs of SDC-2 (NM_013082.2) and SDC-3 (U52825.1) were cloned into the pEGFP-N3 Vector (Clontech, Palo Alto, CA, USA) or pcDNA3.1 Myc-His(-)A (Invitrogen, Darmstadt, Germany) and expressed in COS-7 cells. The successful incorporation in cellular membranes was verified microscopically and via subcellular fractionation. In order to detect the extracellular domains separately, additionally a myc-epitope tag (9E10) was cloned into the Hind III restriction site of SDC-2 (146/150) and Pst I restriction site of SDC-3 (500/504) of the GFP-constructs.

shRNA knock down constructs for SDC-2 and -3 were obtained from OriGene Technologies (Rockville, USA). In order to monitor transfection efficiencies, the respective knock down cassettes, containing the U6 polymerase III promoter and the shRNA-sequence, were sub-cloned into the pGFP-RS shRNA cloning plasmid (OriGene). Knockdown capacities of the individual constructs were analyzed in COS-7 cells expressing the respective SDC containing an internal myc-tag. The two most effective shRNAs were used for all down stream experiments (SDC-2: TI566275 5′-CCAAAGTGGAAACCATGACGTTGAAGACA-3′ and TI566276 5′-GCTACGACCTTGGAGAACGCAAACCATCC-3′; SDC-3 TI566167 5′-AACGCTGGCGCAATGAGAACTTCGAGAGG-3′ and TI566168 5′-AACCTGACA AGCAGGAGGAGTTCTACGCT-3′) and corresponding scrambled controls. For Lentivirus mediated knockdown of SDC-2 and -3 in primary neural cultures, the selected knockdown cassettes from the pGFP-RS shRNA constructs were subcloned into the PacI restriction site of the pFUGW vector [25].

### Lentivirus production

The generation of lentiviruses was done by co-transfecting HEK293T-cells, grown in DMEM+, with the respective shuttle plasmid (pFUGW or pSDC2/3shRNA-FUGW) and two packing plasmids (pSPAX2 and pHCMV-VSVg) using Lipofectamine 2000 (Invitrogen) according to the manufacturers protocol. After 12 hours 60% of the DMEM+ were exchanged against DMEM− in order to reach a final FCS concentration of 4%. 24 hours later the virus-containing medium was collected, briefly centrifuged at 2000 rpm for 5 min and the clear supernatant filtered using a 0.45 µm-filter. Finally, the viruses were spun down in an ultracentrifuge and the remaining virus-containing pellet was gently dissolved in 100 µl NB+, divided in aliquots and stored at −80°C. The viral titer was estimated as infective units via the determination of the GFP-fluorescence in infected HEK293T cells.

### Statistical Analysis

Statistical analyses were performed using Student's t-test.

## Results and Discussion

To test the idea that Y-P30 might regulate the nuclear localization of CASK we applied the peptide for three and six hours to cortical primary neurons at the concentrations of 6 µg/ml that promotes neurite outgrowth [11] and then performed immunocytochemical stainings and quantitative immunoblotting experiments. We found that bath application of the peptide at DIV18 resulted in an increased nuclear accumulation of CASK in comparison to controls. This increase was evident in immunostainings (Fig. 1c) as well as on immunoblots aimed at quantifying CASK protein content in a P1 fraction that contains purified neuronal nuclei (Fig. 1d–f). Surprisingly, however, the opposite effect was observed at DIV8. At this developmental stage supplementation of the culture medium for three or six hours with Y-P30 resulted in significantly reduced nuclear CASK levels (Fig. 1g). The nuclear accumulation of CASK at DIV18 was blocked when we incubated the cultures with Heparitinase I and Chondroitinase A,B,C to remove sugar side chains prior to Y-P30 administration (Fig. 1h+i), a treatment that abolishes binding of Y-P30 and PTN to SDC-2 and -3 [11].

**Figure 2:**
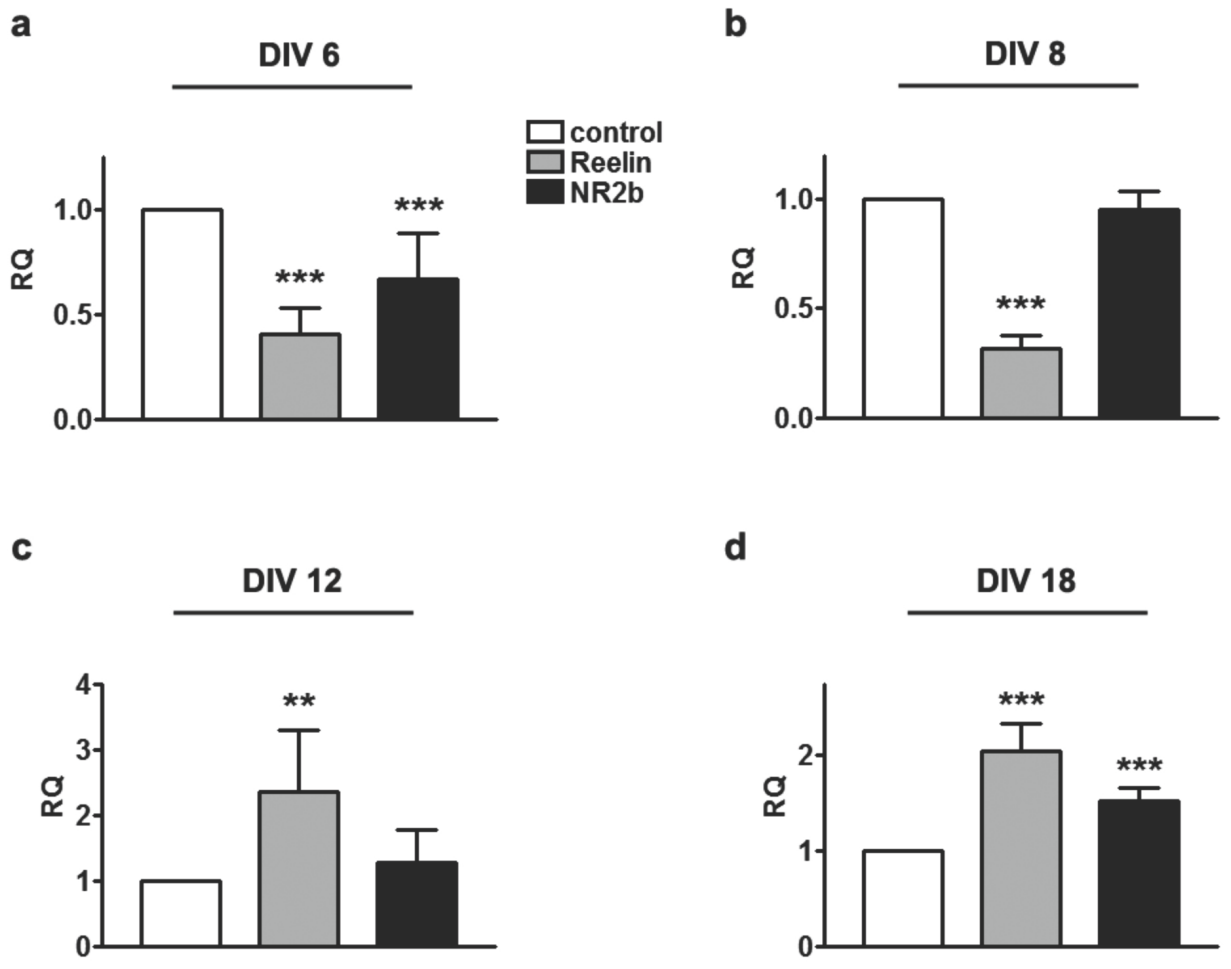
Transcript levels of reelin and GluN2B in primary cortical neurons are altered by Y-P30 depending upon the age of the culture. (a–d) Primary cortical cultures were supplemented with Y-P30 at different developmental stages and harvested after 3 h. Following mRNA extraction and reverse transcription, the samples were analysed using quantitative PCR. In younger neurons, DIV6 (a) and DIV8 (b), a significant decrease of the relative Reelin transcript levels was detected. The GluN2B transcript levels were under these conditions only in cultures at DIV 6 significantly decreased (a). In contrast, Y-P30 administration leads to an accumulation of CASK in the nuclei of more mature neurons and results in an increase of transcript levels of reelin and GluN2B at DIV12 (c) and DIV18 (d). For the analysis of relative quantities (RQ) the reelin and GluN2B data had been normalized with Hprt1 for mRNA variations and were calculated using the 2-ΔΔCt method (Livak and Schmittgen, 2001). Samples were analysed in dupli- or triplicates from 3–7 independent experiments. Black and grey boxes indicate treatment with Y-P30. *** p<0.001, ** p<0.01.

**Figure 3:**
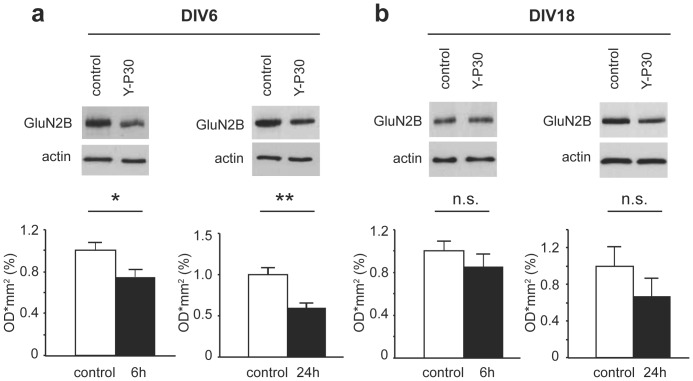
Quantitative immunoblotting of GluN2B levels in cortical primary cultures following Y-P30 application. (a+b) Representative blots probed with a GluN2B or actin antibody are depicted. (a) Protein levels were altered at DIV6 after 6 and 12 h incubation of cortical primary cultures with Y-P30. (b) No significant changes were seen at DIV18. n.s.: not significant. N: 5–7 in each group. ** p<0.01, * p<0.05.

Nuclear CASK is supposed to regulate the expression of TBR1 target genes like reelin or the GluN2B subunit of the NMDA-receptor. GluN2B containing NMDA receptors predominate during early development and synaptogenesis, whereas following synaptic maturation the number of GluN2A-containing receptors increases. In qPCR experiments we found that bath application of Y-P30 indeed regulated the mRNA expression levels of reelin and the GluN2B-subunit. In early development at DIV 6 reelin and GluN2B transcript levels were significantly lower in cultures treated with Y-P30 as compared to control conditions (Fig. 2a). This effect was less apparent at DIV 8 for GluN2B and reversed for reelin at DIV 12 (Fig. 2b+c). Interestingly, at DIV 18 administration of the peptide to the medium induced elevated mRNA levels for both reelin and GluN2B (Fig. 2d). Thus, in young neurons, where Y-P30 reduces the nuclear levels of CASK, also CASK/TBR1 regulated gene expression is reduced while this is no longer found in older cultures. To corroborate these findings we performed quantitative immunoblotting experiments and probed these blots with a GluN2B antibody. We found that at DIV6 GluN2B protein levels were indeed lower in Y-P30 treated cultures (Fig. 3a), whereas at DIV18 bath application of Y-P30 had no significant effect (Fig. 3b).

**Figure 4:**
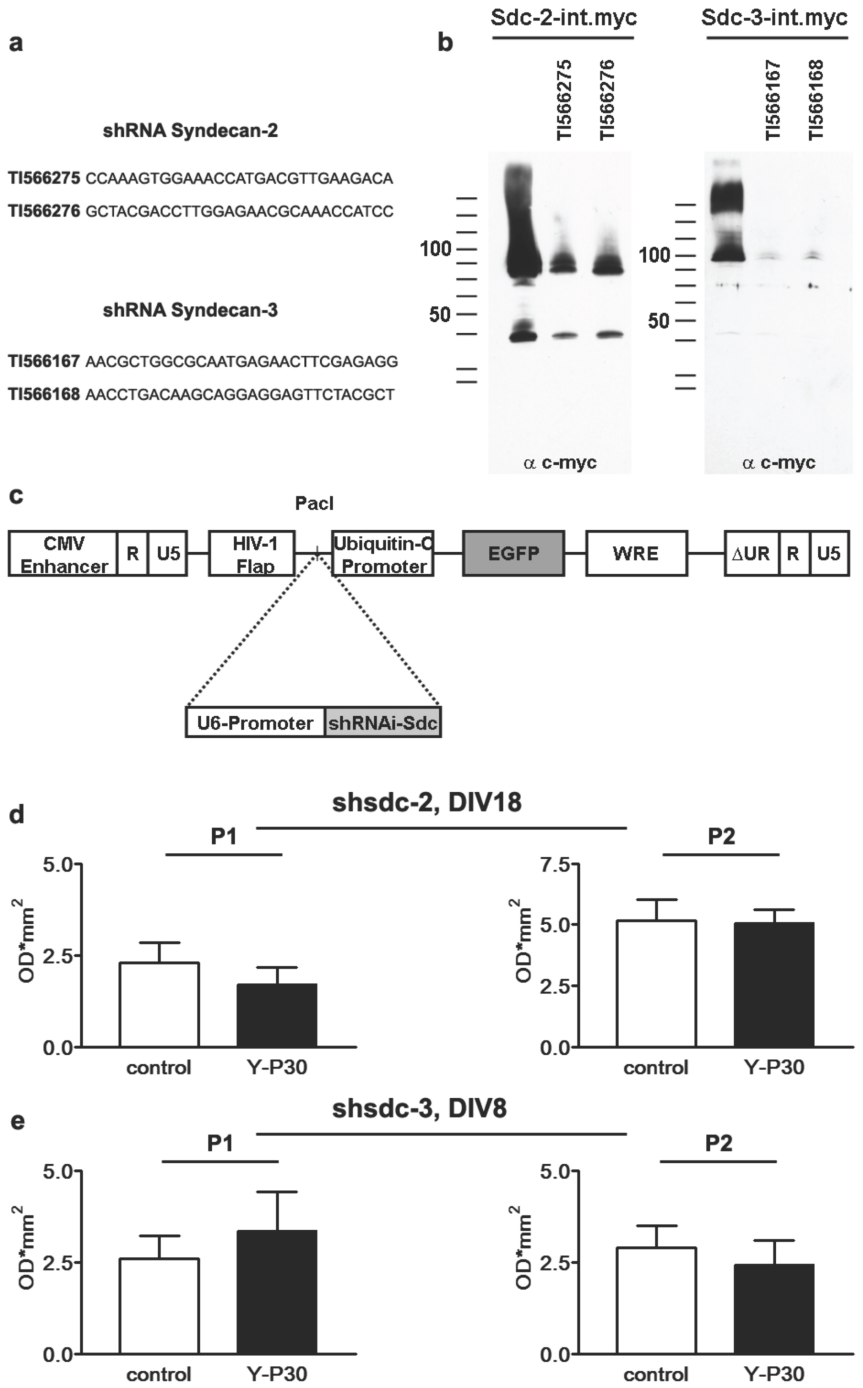
Knock down of SDC-2 and -3 abolishes the Y-P30 induced CASK translocation in primary cortical neurons. (a+b) shRNA knockdown was tested in co-transfected, SDC-2-myc or SDC-3-myc over-expressing Cos7 cells. The knockdown effects of the two most efficient shRNA sequences for each SDC (a) are shown in representative Western-blots (b). Note the efficient knockdown of the respective SDC. Syndecans are glycoproteins and the different bands detected by the myc antibody probably represent differentially glycosylated protein isoforms. (c) In order to establish a Lentivirus-based knockdown system, the appropriate shRNA cassettes of the pRS vectors, containing the U6-polymerase III-promoter, were subcloned into the PacI restriction site of the FUGW vector. (d, e) In mature primary cortical cultures (DIV18), expressing the knockdown construct against SDC-2, the Y-P30 induced CASK translocation is completely abolished. Similar effects after Y-P30 supplementation were obtained in young primary cortical cultures (DIV8) expressing the SDC-3 knockdown construct, indicating different cellular effects of both SDC (n = 4–6).

Taken together these results lead to the question about the underlying mechanism of the differential effect of Y-P30 on the nuclear localization of CASK during neuronal development. We reasoned that this differential effect might be related to the fact that the two family members that are abundant in neurons, SDC-2 and -3, are expressed at different levels during development [16], [26]. SDC-3 is prominently expressed during early development and mainly localized in axons whereas SDC-2 protein levels increase later during synaptogenesis when SDC-2 is mainly found in dendrites and synapses [16], [26]. To address the question whether binding to SDC-2 and -3 mediates the effect of Y-P30 on the nuclear localization of CASK we employed an shRNA approach to knock down both SDC (Fig. 4a+c). Since available SDC antibodies were not found to be suitable for immunostainings we transfected HEK-293T cells with SDC-myc constructs and the corresponding shRNA plasmid and checked for knock down efficiency (Fig. 4b). Thereafter we transfected hippocampal primary neurons with the most efficient shRNA construct for SDC-2 or -3 and compared the effects of Y-P30 application at DIV 8 and 18 to control cultures. We found that knock down of SDC-2 at DIV18 (Fig. 4d) and SDC-3 at DIV8 completely abolished the effect of Y-P30 on the nuclear localization of CASK (Fig. 4e).

**Figure 5:**
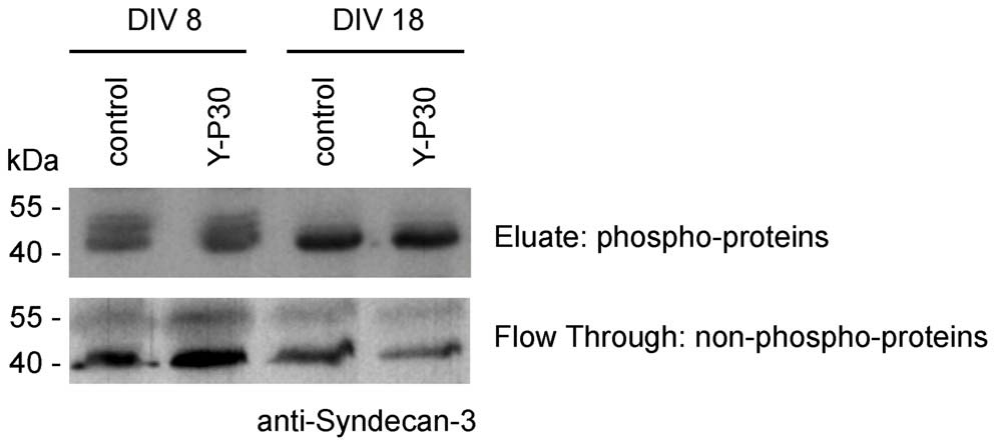
Y-P30 treatment has no apparent effect on SDC-3 phosphorylation. Phosphoproteins were purified by affinity chromatography and the flow though containing non-phosphorylated proteins and the eluate containing phosphorylated proteins were loaded on the gel. Equal amounts of protein were loaded on the affinity columns. The blots were probed with SDC-3 antibody.

It has been reported that SDC-3 is phosphorylated at several sites and that the expression of the phosphorylated form of SDC-3 decreases during neuronal development [27], which might alter its function. We therefore checked for Y-P30 induced phosphorylation of SDC-3 but could not identify apparent differences at DIV8 and DIV18 (Fig. 5).

**Figure 6:**
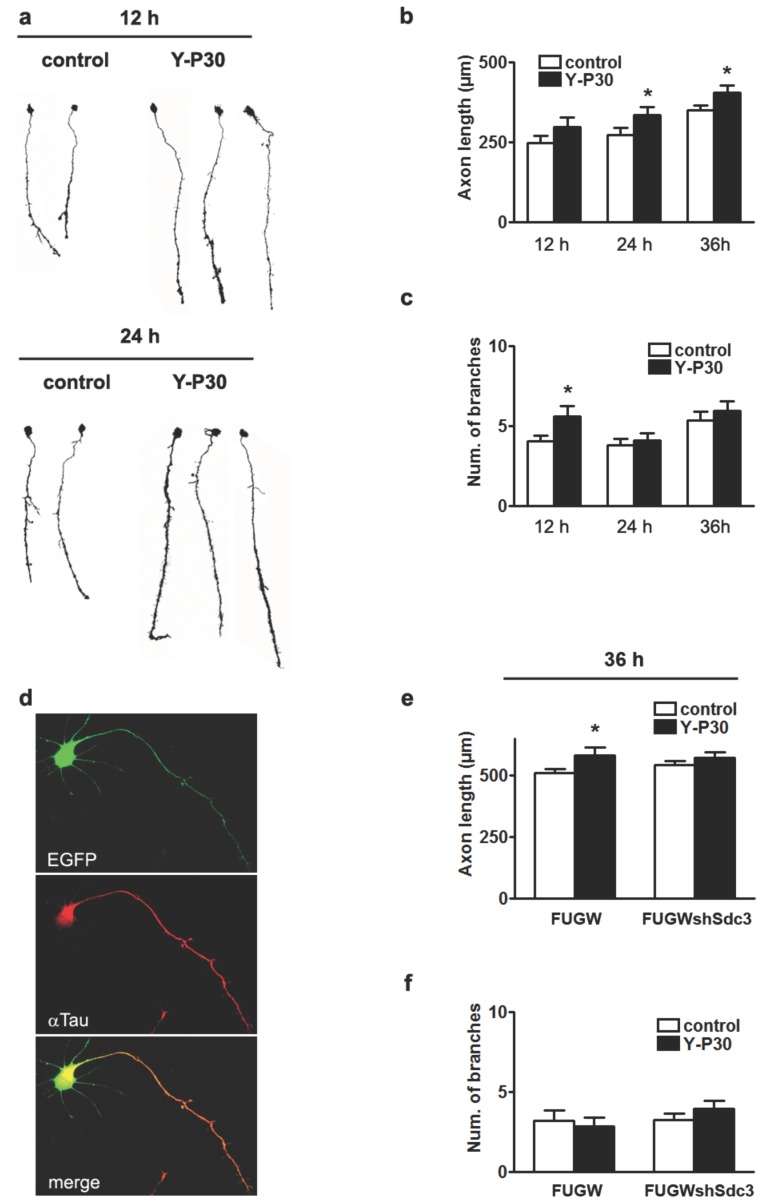
The Y-P30 induced axonal growth and branching in young primary cortical neurons requires SDC-3. (a–c) 24 hours after plating primary cortical neurons were supplemented with 20 µM Y-P30 and finally fixed at 12 h, 24 h and 36 h after treatment. The Y-P30 induced enhancement of axonal growth and partially branching in primary cortical neurons, fixed 12 h and 24 h after supplementation, is depicted as a reconstruction (a). The quantitative analysis is shown in (b) and (c). For SDC-3 knockdown primary cortical cultures were infected immediately after plating with Lentivirus-based shSDC3-FUGW constructs or empty FUGW as a control. After 24 h the infected cultures were supplemented with Y-P30 as described previously and finally fixed 36 h later. An example for a primary cortical neuron expressing the shSDC3-FUGW construct is shown in (d). Scale bar is 20 µm. Quantitative analysis of axon length 36 h after supplementation (e) revealed that the Y-P30 induced axonal growth is significantly abolished in neurons expressing the knockdown construct, but not in neurons expressing FUGW alone. For axonal branching no differences could be detected under these conditions (f). Black boxes indicate treatment with Y-P30. N: 30–40 in each group; * p<0.05.

In previous work we observed that Y-P30 promotes neurite outgrowth of thalamic neurons in primary cultures [11]. We followed up on these results and examined in more detail whether the application of the peptide might selectively stimulate axonal outgrowth of hippocampal and cortical primary neurons in early development. To this end we applied Y-P30 for 24 h or 36 h in cortical cultures 24 h after plating. We then determined the length and branching of the longest neurite (Fig. 6a). Bath application of Y-P30 in cortical cultures resulted in faster extension of axons (Fig. 6b). In addition, the number of branches was increased 12 h after application of the peptide (Fig. 6c).

We next employed a SDC-3 knock down and analyzed axon length and branching in cultures that were treated with Y-P30 at DIV 3 or DIV 6 (Fig. 6d). A SDC-3 knock down had only minor effects on axon outgrowth and branching as compared to non-transfected or scrambled-transfected control cells (Fig. 6e+f). Interestingly, however, the Y-P30 induced axon outgrowth was significantly reduced in cells transfected with the SDC-3 knock down construct (Fig. 6e).

**Figure 7:**
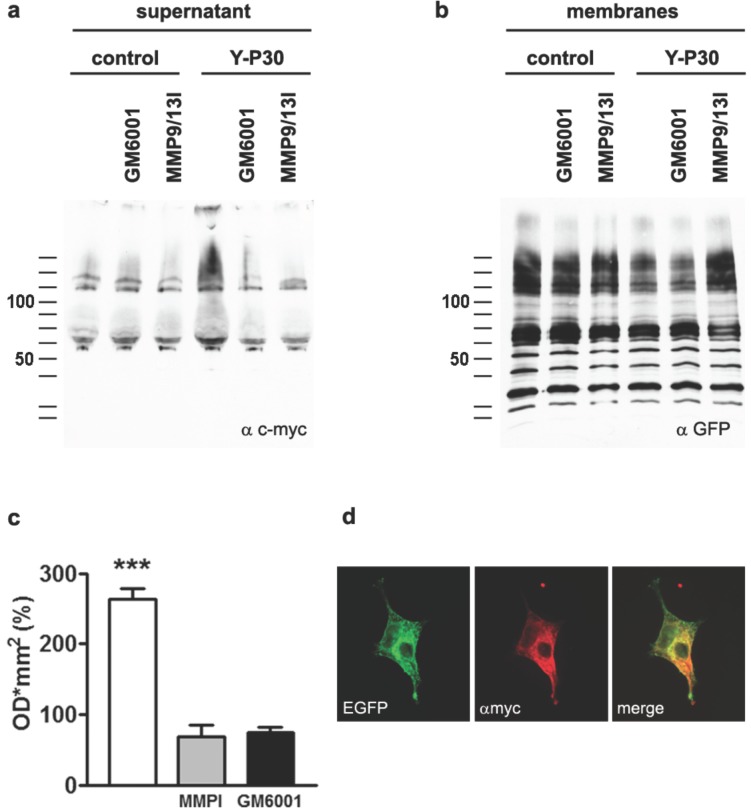
Y-P30 induces the proteolytic cleavage of the extracellular domain of SDC-2. (a) COS7 cells, expressing SDC-2intmyc-GFP, were supplemented with 20 µM Y-P30 or a mock control. For the inhibition of matrix metalloproteinases either 50 nM GM6001 or 20 nM of MMP9/13 inhibitor I were used. After 3 h the culture media were collected, the containing proteins precipitated with ethanol and subsequently analysed with quantitative immunoblotting. In order to analyse the successful over expression and membrane-incorporation of the tagged fusion proteins, the respective cells were harvested, fractionated and the membrane proteins evaluated on western blots. A representative quantitative immunoblot analysis of the SDC-2 ecto-domain from the culture medium is shown in (a). Note that supplementation with Y-P30 increases the amount of the myc-tagged SDC-2 ecto-domain, whereas GM6001 as well as MMP9/13I abolished the Y-P30 dependent cleavage. The total expression and incorporation of the SDC-2 construct was analysed in membranes after subcellular fractionation using western-blot analysis (b). The relative amounts of the detected SDC-2 ecto-domains from the culture media are depicted in (c) as % to the control. N: 3–6; *** p<0.0001. (d) Illustrates an immunofluorescence image of the SDC-2intmyc-GFP expression in COS7 cells, showing a clear merge of the GFP-fluorescence from the C-terminus of the fusion protein and the myc-tag, incorporated into the ecto-domain of SDC-2. Scale bar is 20 µm.

These data suggest that the effects of Y-P30 on axon outgrowth as well as on the nuclear localization of CASK depend upon the expression of SDC-2 and -3. However, it is puzzling that binding to SDC-3 during early development and to SDC-2 during synaptogenesis has fundamentally different effects on the nuclear localization of CASK and CASK/TBR-1 mediated gene expression. We therefore asked next what might be the underlying mechanism and reasoned that it might a differential shedding of extracellular domain of SDC-2 as compared to SDC-3. Previous work has shown that SDC-3 is susceptible to intramembranous cleavage by γ-secretase [28] and that Y-P30 has proteolytical activity on its own [29]. We therefore overexpressed SDC-2 and -3 that harbor a myc-tag in their extracellular domain in HEK-293 cells and examined cleavage of the extracellular domain after application of Y-P30. It turned out that Y-P30 induced cleavage of the extracellular domain of SDC-2 (Fig. 7a) but not of SDC-3 (data not shown). An inhibitor of Matrix-metalloproteinase 9 (MMP9) blocked the effects of Y-P30 on shedding of the extracellular domain of SDC-2, suggesting that proteolytical activity of Y-P30 alone is not sufficient to induce cleavage (Fig. 7).

In previous work we found that the neuritogenic effects of Y-P30 are based on binding of the peptide to PTN and SDC-2 and -3 [11]. In the present study we have analyzed potential mechanisms by which the interaction of Y-P30/PTN with SDC can regulate neurite outgrowth. We found that during early development Y-P30 application in primary neurons reduces the nuclear localization of CASK whereas the opposite was found in older neurons. This effect of Y-P30 needs the association with SDC-3 in young and SDC-2 in older neurons. The underlying signaling mechanism probably involves the nuclear localization of CASK and a differential effect of Y-P30 on ectodomain cleavage of SDC-2 and -3. In young cultures Y-P30 binding appears to reduce ectodomain shedding of SDC-3 and thereby probably stabilizes a SDC-3/CASK complex that shifts the distribution of CASK from the nucleus to the plasma membrane. In older neurons Y-P30 binding to SDC-2 has the opposite effect and intramembranous cleavage of SDC-2 might release CASK for nuclear import.

One interesting question regarding these actions of Y-P30 concerns the mechanism of SDC cleavage. Ecodomain shedding of SDC has been described for all family members in different tissues and is considered to be important for SDC signalling [30]. In many cases matrix metalloproteases are involved in ectodomain shedding [31], [32] but only few co-factors like insulin [33] have been shown to be involved in this cleavage event. It is tempting to speculate that following shedding intramembranous cleavage of SDC-3 by γ-secretase [28] might release the intracellular domain. In turn this would allow for nuclear transport of CASK [17], [26]. Unfortunately very little is known about the mechanisms of transport to the nucleus and whether transport is regulated by ligand binding to SDC. Nuclear CASK associates with TBR-1 and this transcription factor has been shown to regulate the expression of genes important during neuronal development [26], [34]. In line with this role we found increased expression of reelin and GluN2B mRNA at cortical neurons at DIV 18. In summary, we propose that in development the neuritogenic effects of the peptide as well as its effect on gene expression might be related to SDC-binding and the control of nuclear import of CASK.

Apart from Y-P30 the only other known soluble ligand of SDC is the ubiquitously expressed trophic factor PTN. It was shown that the SDC interaction with CASK depends on SDC homodimerization [35]. This is interesting since Y-P30/PTN profoundly oligomerize and subsequent SDC binding might ease clustering and homo-dimerization, which in turn might affect signaling. The signaling role of PTN is not well investigated. We speculate that the presence of PTN might be necessary for the effects of Y-P30 on ectodomain shedding or prevention of SDC cleavage. The association of both peptides with SDC might occur also in other tissues and with other family members like SDC-1 and -4. Along these lines it was indeed shown that Y-P30 is expressed in breast cancer and that ectodomain shedding of SDC might be causally related to a poor prognosis in this and potentially other types of cancer [36], [37].

At present it is unclear whether the Y-P30 peptide might play a role during neuronal development in human brain. The expression of Y-P30 mRNA has been reported in human brain regions [36]. However, recent reports indicate that the human Y-P30/dermcidin peptide as well as the mRNA are extremely stable for several weeks and can be used as abundant markers for human sweat in forensic medicine [38]. Thus, the contamination of cell cultures and tissue sections during processing by human skin in analogy to keratin is a serious concern in all studies looking at Y-P30 expression. In light of the difficulties to identify a rodent Y-P30/dermcidin gene it is therefore conceivable that reports by us [2] and potentially also of others on the presence of the peptide in rodents are confounded by contaminations with the human peptide.

## Conclusion

The survival promoting peptide Y-P30 promotes neurite outgrowth in a manner that involves SDC/Cask signaling. Interference with the nuclear localization of CASK using the peptide might be of interest for pharmacological applications to promote neurite outgrowth and cell survival.
